# Real-time quantitation of thyroidal radioiodine uptake in thyroid disease with monitoring by a collar detection device

**DOI:** 10.1038/s41598-021-97408-y

**Published:** 2021-09-16

**Authors:** Prasanna Santhanam, Lilja Solnes, Tanmay Nath, Jean-Paul Roussin, David Gray, Eric Frey, George Sgouros, Paul W. Ladenson

**Affiliations:** 1 Division of Endocrinology, Diabetes, and Metabolism, Department of Medicine, Johns Hopkins University School of Medicine, 1830 E. Monument St./Ste. 333, Baltimore, MD 21287 USA; 2 Department of Radiology and Radiological Science, Johns Hopkins University School of Medicine, Baltimore, MD 21287 USA; 3 Department of Biostatistics, Bloomberg School of Public Health, Johns Hopkins University, Baltimore, MD 21287 USA; 4 AG Medical Company, Saint Aubin, France

**Keywords:** Endocrine cancer, Thyroid diseases, Biomedical engineering, Electrical and electronic engineering

## Abstract

Radioactive iodine (RAI) is safe and effective in most patients with hyperthyroidism but not all individuals are cured by the first dose, and most develop post-RAI hypothyroidism. Postoperative RAI therapy for remnant ablation is successful in 80–90% of thyroid cancer patients and sometimes induces remission of nonresectable cervical and/or distant metastatic disease but the effective tumor dose is usually not precisely known and must be moderated to avoid short- and long-term adverse effects on other tissues. The Collar Therapy Indicator (COTI) is a radiation detection device embedded in a cloth collar secured around the patient’s neck and connected to a recording and data transmission box. In previously published experience, the data can be collected at multiple time points, reflecting local cervical RAI exposure and correlating well with conventional methods. We evaluated the real-time uptake of RAI in patients with hyperthyroid Graves’ disease and thyroid cancer. We performed a pilot feasibility prospective study. Data were analyzed using R^©^ (version 4.0.3, The R Foundation for Statistical Computing, 2020), and Python (version 3.6, Matplotlib version 3.0.3). The COTI was able to provide a quantitative temporal pattern of uptake within the thyroid in persons with Graves’ disease and lateralized the remnant tissue in persons with thyroid cancer. The study has demonstrated that the portable collar radiation detection device outside of a healthcare facility is accurate and feasible for use after administration of RAI for diagnostic studies and therapy to provide a complete collection of fractional target radioactivity data compared to that traditionally acquired with clinic-based measurements at one or two time-points.

*Clinical Trials Registration* NCT03517579, DOR 5/7/2018.

## Introduction

For 80 years, radioactive iodine (RAI) has been a key therapeutic modality for certain forms of hyperthyroidism and differentiated thyroid cancer (DTC)^1,2^. The optimal administered I-131 doses for these conditions have generally been determined either empirically or with the support of crude dosimetry based on an initial I-123 or I-131 tracer dose and follow-up fractional uptake observations over the therapeutic target(s) at one or two subsequent times. The tracer doses also differ for I-123 (7.4–14.8 MBq [200–400 μCi]) and I-131(37–74 MBq [2 mCi]) diagnostic scanning^3^. Although RAI is safe and effective in most patients with hyperthyroidism, not all individuals are cured by the first dose, and most develop post-RAI hypothyroidism^1,2^. The success percentage for RAI therapy and the development of post ablative RAI is approximately 80–90%^1,2^. Similarly, in thyroid cancer patients, RAI is safe and highly effective in ablating remnant normal thyroid tissue. However, it only sometimes induces remission of remaining nonresectable cervical and/or distant metastatic disease because the effective tumor dose is usually not precisely known and must be moderated to avoid short- and long-term adverse effects on other tissues. An important limitation of the crude dosimetry, currently employed to support RAI dose determination, has been the limited number of time points at which the tracer dose’s extent of lesional concentration is obtained due to logistical factors.

The Collar Therapy Indicator (COTI) is a radiation detection device embedded in a cloth collar secured around the patient’s neck and connected to a recording and data transmission box. In previously published experience, the data can be collected either at multiple time points, reflecting local thyroidal residual tissue RAI exposure and correlating well with conventional methods^4^.

In one previously studied proof of principle study, there was a good correlation between COTI measured I-131 uptake and the standard gamma probe at 2-h and 24-h time points, though the 24-h time point was much better^5^.

Thus, the device’s ability to collect, store, and report from outside the healthcare setting (it can be safely used at home by the patient) permits a complete estimation of lesional activity over time and a more precise prediction of the therapeutic radiation dose that will be delivered with subsequent I-131 administration. This study evaluated the real-time uptake of RAI in persons with hyperthyroid Graves disease and thyroid cancer.

However, the actual therapeutic dose decisions in this pilot trial were not based on the collar device measurements. Patients with Graves disease received 6.67–7.4 MBq (180–200 µCi) I-131 per gram of estimated gland mass based on the conventional method of dose calculation, based on a 24-h % uptake and gland volume. Persons with thyroid cancer received the standard dose for remnant ablation prior to the placement of the COTI device, as per the American Thyroid Association (ATA) guidelines^6^.

## Research design and methods

### Primary objective

To perform a pilot (exploratory) study using a novel AG Medical Collar Therapy Indicator (COTI) to evaluate the variability in radioiodine kinetics across patients treated for hyperthyroid Graves disease and postoperative thyroid remnant ablation for thyroid cancer.

Secondary Objectives;

- To validate the use of the cervical collar device as an enhancement for quantitative measurement of thyroid uptake in patients after diagnostic I-123 or I-131 and therapeutic I-131 in Graves disease
- To validate the use of the cervical collar device as an enhancement for quantitative measurement of thyroid uptake in patients after therapeutic I-131 for thyroid remnant ablation in thyroid cancer.
- To obtain detailed early-phase thyroid uptake data using a cervical collar device in Graves disease and thyroid cancer patients as described above.

It was a prospective pilot feasibility clinical trial involving patients of both hyperthyroidism and thyroid cancer. The COTI device has been conceptualized and developed by the AG Medical company, Saint Aubin, France (ag-medical.com). The study was approved by the Johns Hopkins institutional review board after a detailed evaluation of the risks and benefits of the device. Johns Hopkins Engineering Services examined the device and deemed it to be of low/minimal risk. The study was registered at clinicaltrials.gov (NCT03517579, Registration date 5/7/2018, Start date 12/11/2018, and presently ongoing). Informed consent was obtained after detailing the risks and benefits of the device in simple terms from all participants and their legal guardians. The Johns Hopkins IRB and ethics committee approved the research, and the research was performed in accordance with relevant guidelines/regulations. The level of radiation exposure of SPECT/ CT scans and the risks and benefits associated with the imaging procedures were clearly documented for the participants’ understanding. (Please see supplementary material for details on the protocol and the consent).

### Study device

COTI stands for *Collar Therapy Indicator* and is a non-invasive medical device that indicates gamma radiation activities taken from patients undergoing I-131 treatment for thyroid diseases like thyroid cancer and hyperthyroidism. COTI patients undergoing RAI treatment are typically maintained in isolation to limit radiation exposure to the hospital support staff. COTI is a lightweight, battery-powered wearable device that collects and displays gamma radiation (indicates counts-per-second) to a remote computer tablet via wireless communication. The information collected by the tablet may be used to indicate rising or falling radioactivity levels (counts-per-second) at various points on the patient.

COTI consists of three main elements:

1. Detector Modules that count the gamma radiation activity in counts-per-second (Upto 80,000 counts per second, energy window of 60–600 keV). The device uses silicon photomultipliers (SiPMs) with multiple micron-level avalanche diode elements, approximately measuring 20–30 μm with amplification of 10^6^, comparable to the regular photomultiplier tube^5^. The LoHI Detector used in the study has four 6 × 6 mm SiPMs with four 5 mm CsI (Tl) crystals with a max sensitivity variability of 5% between two detectors and a photon accumulation time of 1 s, and uncertainty of < 2%.
2. A Control Unit that powers and communicates with the Detector modules and relays the information to the remote Tablet.
3. A Tablet with software that receives input from the Control Unit and collects data that may be exported. A smartphone is given to the patient along with the CoTI® Control Unit and Detectors in another configuration. Each COTI Detector module has the same essential function. The different versions relate to the level of radioactivity used are shown (Table 1).

**Table 1:** The different detectors and their sensitivities.

Detector module	Thyroid activity		Comments
	(MBq)	(mCi)	
LoHi	0.02–30	0.0005–0.8	Low-dose uptake
Standard	1–600	0.027–16.2	Normal uptake & therapy
Attenuator	5–1000	0.135–27	High-dose therapy
Collimator	1–600	0.027–16.2	Research

A low-activity collar is employed for diagnostic I-123/I-131 (7.4–44.4 MBq [0.2–1.2 mCi] I-123/I-131) imaging and uptake quantification for hyperthyroid and thyroid cancer patients. After therapeutic I-131 (1110–7400 [30–200 mCi] MBq I-131), a medium-activity collar is employed for dose measurement in thyroid cancer patients. Our study used the LoHi detectors for the diagnostic I-123 scans and the Standard detector for the I-131scan (in the second thyroid cancer patient).

The eligibility criteria were as follows:

Inclusion criteria;

- Patients with Graves disease confirmed by laboratory testing.
- Patients with intermediate and high-risk differentiated thyroid cancer require radioiodine remnant ablation or moderately high dose I-131 to treat residual cervical disease.
- Patients were getting care at the Johns Hopkins Hospital- Main campus as well as Bayview Hospital.
- Persons were able to understand simple English and able to follow instructions.

Exclusion Criteria;

- Patients with diseases involving cervical spine, such as spondylosis and severe degenerative joint disease.
- Patients with a planned or ongoing pregnancy.

The initial two patients had hyperthyroid Graves disease confirmed by laboratory testing. The subsequent two patients had intermediate and high-risk DTC requiring thyroid remnant ablation and treatment of residual cervical disease. Patients with cervical spine diseases, such as spondylosis and severe degenerative joint disease, were excluded. The enrolled patients in our study were young and technologically competent and did not have comorbidities like congestive heart failure or chronic kidney disease.

The patients with hyperthyroidism (patients 1 and 2) and DTC (patients 3 and 4) were provided with detailed instructions and training regarding using the COTI device. They were directed to place the front of the collar containing the radiation detection device (between the hyoid bone and the suprasternal notch). In addition, they were told to ensure the correct orientation of the two detectors over the right and left thyroid lobes (if having hyperthyroidism) and on either side of their thyroidectomy scar in the case of DTC. A 2-h pre-study trial recording the detection data was done to ensure accurate location of the device, effective radiation detection and transmission, and comfort of use. The patients were then instructed to take measurements over 10 s, 15–50 times daily during their waking hours. The measurements were relayed to the tablet through Bluetooth and via local Wifi and the Internet a secure server. This ensured participant compliance and also provided us with a mechanism to track the readings in real-time. If there was any technical issue, a contact number was provided for troubleshooting.

Detailed information was obtained about the patients’ history of hyperthyroidism or DTC thyroid, including previous laboratory test result\s, imaging findings, and management. Radiologists reporting the scans were blinded to the findings of the COTI.

## Imaging

### I-123 scans

Medications interfering with iodine handling were discontinued. Patients with hyperthyroidism were imaged 4 h after diagnostic I-123 dose administration (3.7–14.8 MBq [100–400 µCi]) using a gamma camera with a pinhole collimator (20% window centered around at 159 keV) in the supine position with an extended neck as per standard procedures^7^. Patients with DTC were imaged 24 h after I-123 administration (74 MBq [2 mCi]). The whole body was scanned with spot views of the head, neck, and mediastinum using a medium energy parallel-hole collimator with a 20% window centered at 364 keV per standard protocol^7^.

### I-131 scan

Seven days after I-131 therapy, planar imaged were acquired with a high-energy collimator (photopeak 364 keV) as per standard protocol^8^. Hybrid SPECT/CT images at the level of the thyroid bed were acquired on a Philips 16-slice camera. Follow-up fused axial SPECT/CT images were obtained at the level of thyroid bed and regions of interest in the head, neck, and chest. Provision for additional SPECT/- CT scans was included in the protocol but deferred based on clinical necessity and risk of radiation exposure.

The device’s detectors were wholly saturated in our second DTC patient during specific measurements as a result of the refractory state of the crystals (after a relatively high I-131 exposure), so those readings were excluded from the analysis. After that, participants were instructed to take the readings after waiting for 1–2 h to restore the responsiveness of the detectors.

## Data analysis

Data from the tablet were downloaded, extracted, cleaned using R^©^ (version 4.0.3, The R Foundation for Statistical Computing, 2020), and analyzed using the following R packages: ggplot2 and ggfittext ggplotify, ggimage, gridGraphics (compatible with version 4.0.3), and Python (version 3.6, Matplotlib version 3.0.3). All the readings showing “saturated” values on the COTI were disregarded. They were first replaced by NA and then removed through application of the appropriate R code.

## Results

The patient characteristics and interpretations of their scan images are shown in Tables 2 and 3.

**Table 2:** Characteristics of Patients with Graves Disease. *TSI *thyroid stimulating immunoglobulins.

ID	Age	Gender	Laboratory Tests	I-123 Uptake	I-123 Scan
Patient 1	51	Female	T3 (Triiodothyronine) 3.69 (0.8–2.0) TSH < 0.01 (0.36–3.74) Free T4 2.0 (0.8–1.5) TSI > 700 (< 140% baseline)	61%(4 h)	Diffuse homogenous uptake without focal nodularity (Fig. 1a)
Patient 2	45	Female	T3 (Triiodothyronine) 6.5 (0.8–2.0) TSH < 0.01 (0.36–3.74) Free T4 8.0(0.8–1.5) TSI > 639 (< 140% baseline)	6-h uptake- 90.6% 24-h uptake—66.3%	Diffuse radiotracer uptake in the thyroid gland (Fig. 2a)

**Table 3:** -Shows the baseline characteristics of the two patients with thyroid cancer.

ID	Age	Gender	Diagnosis	Stage	Diagnostic I-123 scan	Administered RAI dose	Post-treatment I-131 scan
Patient 3	33	Male	Papillary Thyroid Cancer (2.3 cm with vascular invasion involving multiple lymph nodes in the right central neck)	pT2N1aMx	Multiple foci of uptake in the neck 3–4 smaller foci in the Right Lateral Neck A small focus in the Right supraclavicular area (24 h uptake of 0.629%) (Fig. 3a)	75.2 m CI	Largest foci of intense uptake in the Central Neck 3–4 smaller area in the RLN A focus of Uptake in the Right Supraclavicular Area 1–2 small mediastinal foci of increased uptake (Fig. 3c shows planar image and the SPECT/CT)
Patient 4	49	Male	Hurthle Cell Carcinoma (4.4 cm, encapsulated, Angioinvasive (5 vessels))	pT3N0Mx	Two foci of intense uptake in the left neck Medial-Uptake in the thyroid remnant Lateral-Uptake in the Cervical Lymph Node Additional small foci seen within the right neck, superior and lateral to the left thyroid bed(Fig. 4a)	49.7 m CI	Radiotracer Uptake localized in the Left thyroid bed (Fig. 4c shows planar image and the SPECT/CT)

**Figure 1:**
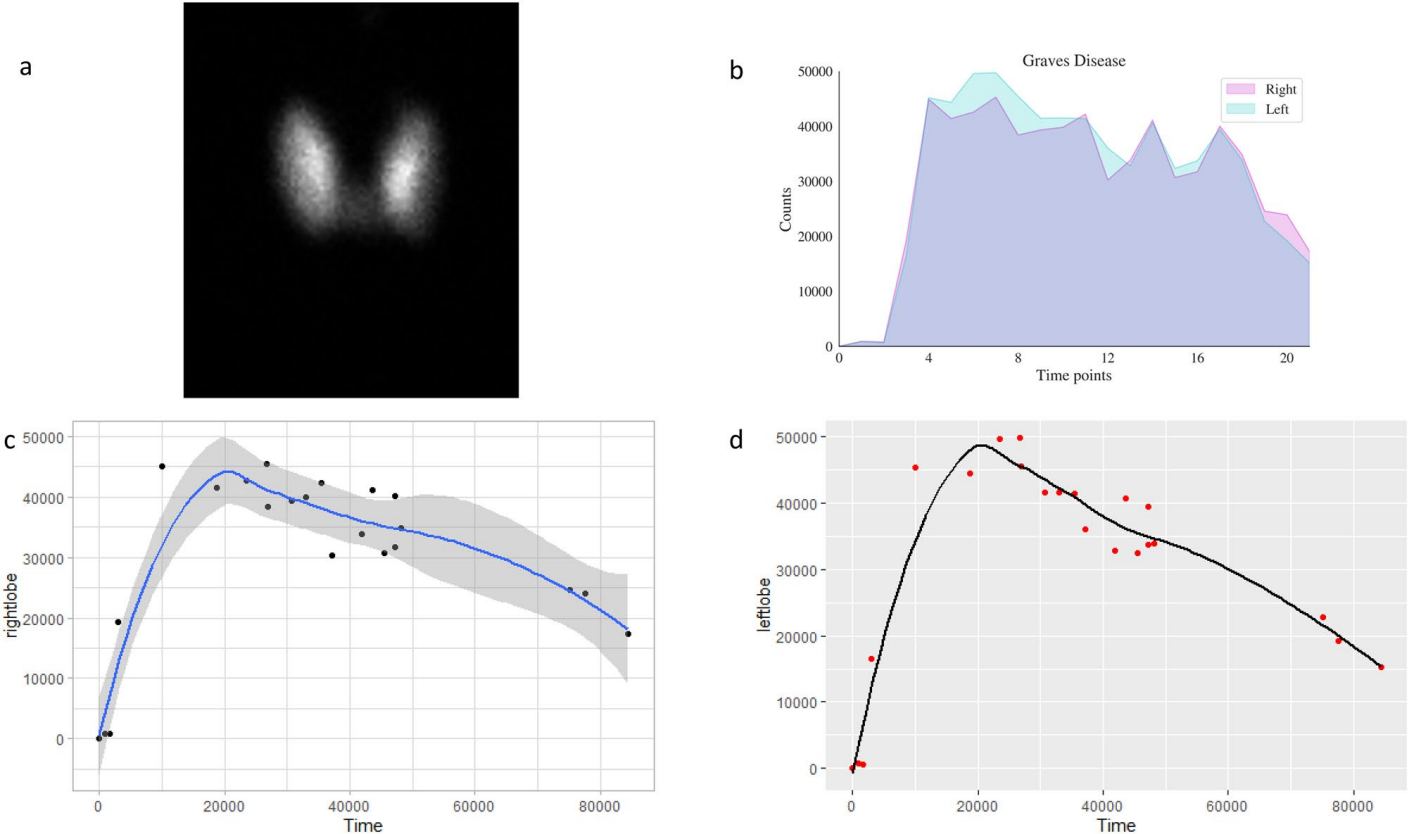
(Graves Patient 1) (**a**) 123 scan showing diffuse heterogenous uptake in both the lobes (**b**) superimposed uptake in the right and left side showing near similarity (**c**) Uptake (absolute counts) in the right lobe with counts and trend over time (in seconds) (**d**) Uptake (absolute counts) in the left lobe with counts and trend over time (in seconds) (loess curves are shown above).

**Figure 2:**
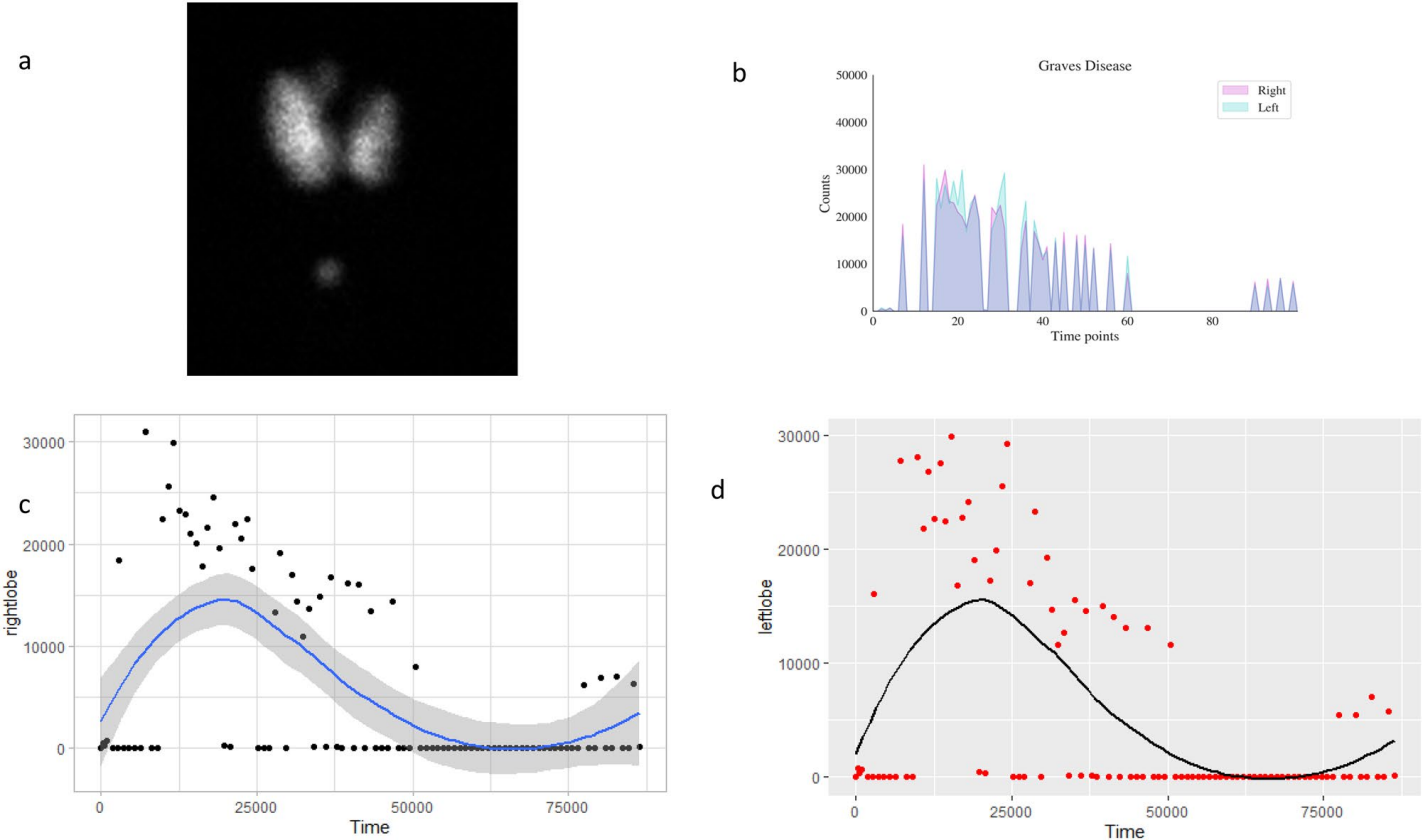
(Graves Patient 2) (**a**) 123 scan showing diffuse heterogenous uptake in both the lobes (**b**) superimposed uptake in the right and left side showing near similarity but with different pattern of uptake (**c**) Uptake (absolute counts) in the right lobe with counts and trend over time (in seconds) (**d**) Uptake (absolute counts) in the left lobe with counts and trend over time (in seconds) (loess curves are shown above).

Hyperthyroid patient 1’s I-123 scan showed a homogenous distribution of activity (Fig. 1a). RAI activity recorded by COTI revealed persistent high uptake in both the lobes over multiple time points (Fig. 1b–d). Hyperthyroid patient 2’s I-123 scan showed (Fig. 2a) a similar homogenous distribution of activity in both lobes; but had a high initial fractional uptake followed by a decrescendo of counts over the course of 24 h in both thyroid lobes (Fig. 2b–d.

In the patients with DTC, I-123 fractional up and distribution were proportional to the sizes of the remnants in the two sides of the thyroid bed.

**Figure 3:**
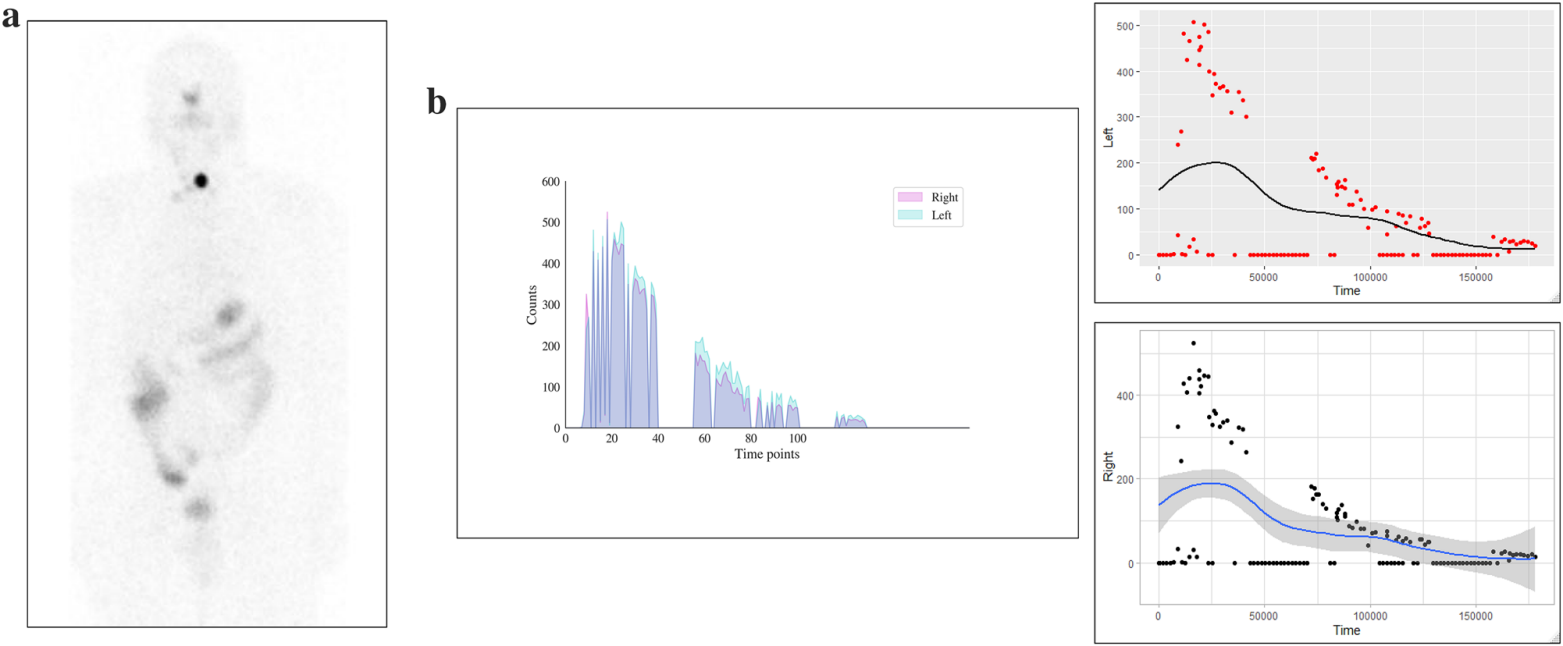

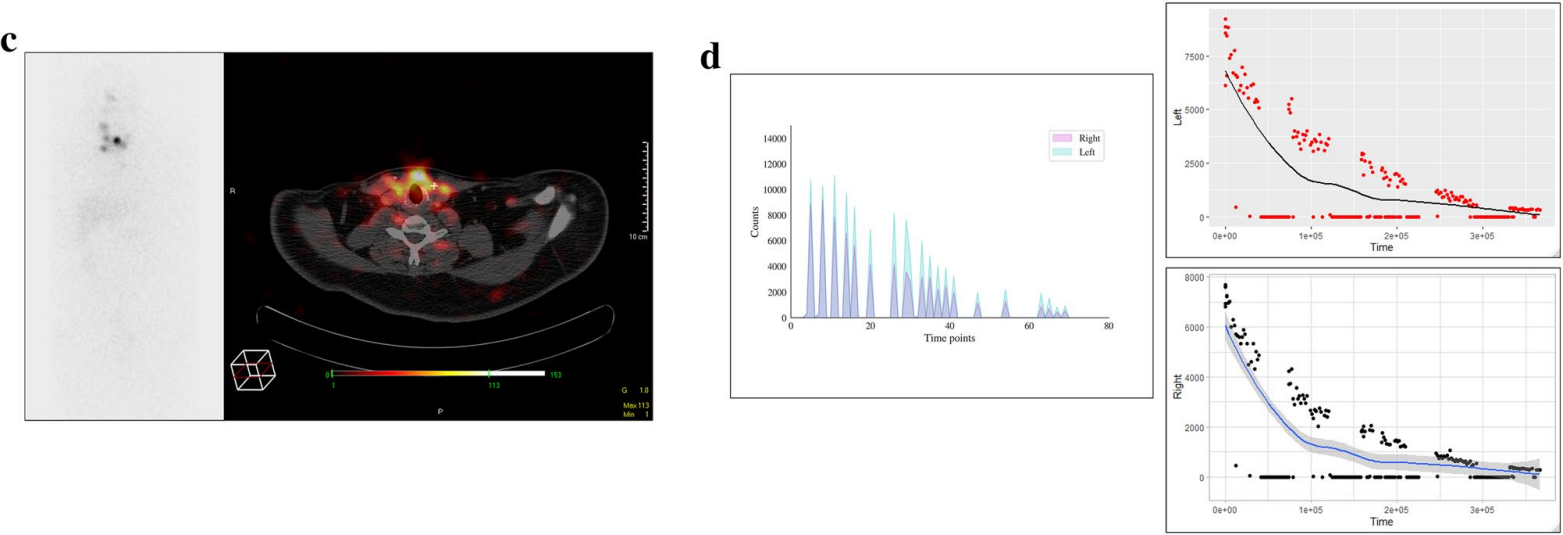
(**a**) First DTC Patient (Patient 3) Whole body planar image showing intense uptake in the neck at the site of remnant and physiological uptake in the bowel, nasopharynx and bladder. (**b**) First DTC Patient (Patient 3)-I-123 COTI measurements : *Left-* Superimposed right and left side over time points, *right upper-* left side COTI measurements over time in seconds*, right lower—*right side COTI measurements over time in seconds. (**c**) First DTC Patient (Patient 3): *Left—*Post-131 planar image, *Right*-Post-131 SPECT/CT showing bilateral areas of intense uptake. (**d**) First DTC Patient (Patient 3)-I-131 COTI measurements : *Left-* Superimposed right and left side over time points, *right upper-* left side COTI measurements over time in seconds*, right lower—*right side COTI measurements over time in seconds.

**Figure 4:**
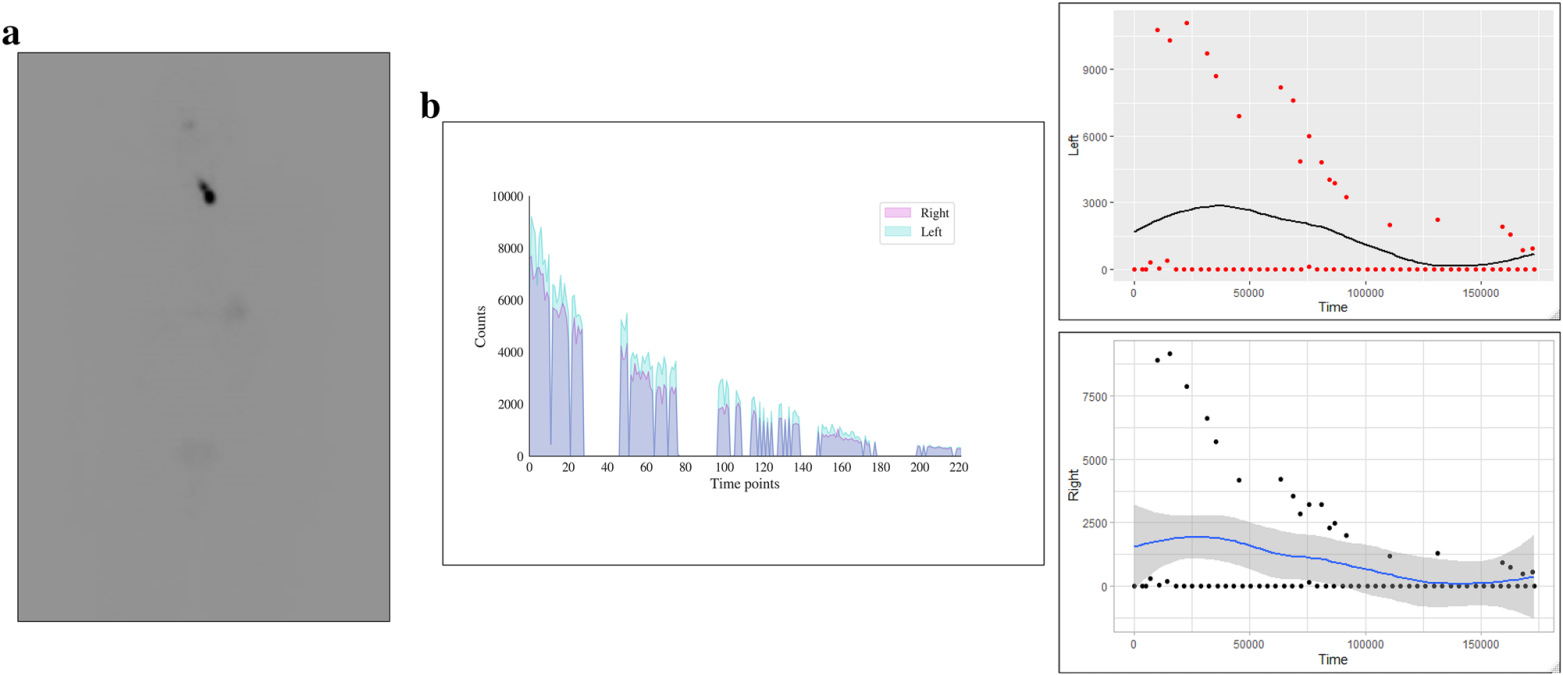

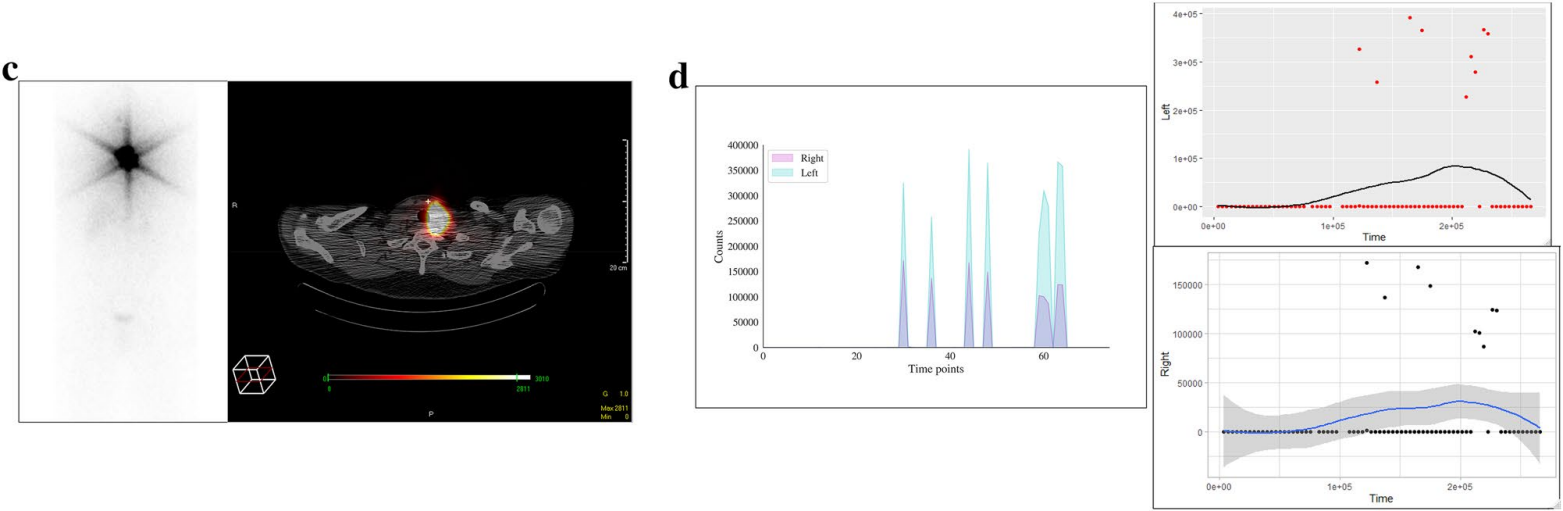
(**a**) Second DTC Patient (Patient 4) Whole body planar image showing intense uptake in the neck at the site of remnant on the left side with extension medially with suppression of the rest of the body. (**b**) Second DTC Patient (Patient 4)-I-123 COTI measurements : *Left-* Superimposed right and left side over time points, *right upper-* left side COTI measurements over time in seconds*, right lower—*right side COTI measurements over time in seconds(absolute counts shown). (**c**) Second DTC Patient (Patient 4): *Left—*Post-131 planar image, *Right*-Post-131 SPECT/CT showing left sided intense uptake with minimal uptake on the right. (**d**) Second DTC Patient (Patient 4)-I-131 COTI measurements : *Left-* Superimposed right and left side over time points(high uptake in the left compared to right), *right upper—*left side COTI measurements over time in seconds*, right lower—*right side COTI measurements over time in seconds (left sided uptakes are significantly higher as seen by the scaling in the Y axis).

In DTC patient 3, the diagnostic I-123 scan showed multiple foci bilaterally in the central neck (Fig. 3a), corresponding to the COTI-detected activity patterns (Fig. 3b). This patient’s I-131 post-treatment scan also showed higher activity in the left neck and multiple foci of low uptake in the right neck (Fig. 3c), which was reflected in higher COTI-detected counts in the left compared to the right neck (Fig. 3d). DTC patient 4 had a large thyroid remnant in the left neck on the I-123 scan (Fig. 4a), corresponding with greater COTI-detected activity on the left (Fig. 4b). This patient’s post-treatment I-131 scan showed much greater uptake on the left, causing a significant star effect (Fig. 4c). In addition, the COTI-measured activity was much higher on the left (Fig. 4d), which could not be fully recorded due to a saturation effect.

## Discussion

This study demonstrates that this portable collar radiation detection device outside of a healthcare facility is accurate and feasible after administration of RAI for diagnostic studies and therapy to provide a complete collection of fractional target radioactivity data compared to that traditionally acquired with clinic-based measurements at one or two time-points. This fuller depiction of the “area under the curve” (after applying appropriate integral calculus equations adjusting for varying time intervals) permits a more accurate prediction of thyroidal radioiodine exposure in treating patients with hyperthyroidism and ablating remnant thyroid tissue in patients with DTC. In doing so, it may be a tool that could facilitate more effective and personalized RAI therapy for these indications with fewer adverse effects.

Previous work on COTI has involved demonstrations of the technique's feasibility and comparability with the standard uptake probe. In the study by Brinks et al., reproducibility, linearity, and accuracy measurements using both I-123 and I-131 radiotracer were performed^4^. In that initial study, the repeated measurements by a phantom (for I-123) showed a mean sensitivity of 2.2 × 102 (± 15) cps/MBq with a good precision of 7%^4^. The coefficient of determination between the COTI measured values and the standard gamma probe was 0.87, confirming the excellent correlation between the two methods^4^. For I-131, there was an uncertainty of 3%, and a correction factor above 2000 counts per second was recommended. In another study on 89 patients (using I-131 at the 2-h and the 24 h time points), COTI uptake values were lower at 2 h but higher at 24 h but with a very good correlation (R^2^ = 0.71 and 0.91 respectively)^5^. Overall, COTI did have excellent agreement with the standard probes.

Imprecision of RAI therapy for hyperthyroidism, with treatment failures (in part due to previously undetected rapid RAI turnover by the gland) and post ablative hypothyroidism are essential concerns in nuclear thyroidology^9^. In a study involving more than 380 patients, thyroid volume, the ratio of the 5-an hour and 24-h RAI uptakes, and the dose of RAI administration were significant determinants of both over and undertreatment of hyperthyroidism^9^.

Various techniques have been employed to estimate the administered dose of I-131 for optimal therapeutic benefit^10,11^. Estimated exposure has typically been extrapolated from the results of activity measurements at one or two time points, e.g., at 4 and 24 h^11,12^. We now know that treating hyperthyroid Graves’ disease with these methods leads to a 13–25% rate of failure to cure hyperthyroidism and a 46–80% rate of long-term hypothyroidism in cured patients^9,13,14^. Similarly, such relatively crude dosimetry doubtless results in significant overtreatment in the case of remnant ablation for differentiated thyroid cancer ^15^. On the other hand, better dosimetry for remnant ablation may permit many patients to be successfully treated with less than 30 mci.

Prior work with COTI has only involved hyperthyroidism but has never been attempted with thyroid cancer remnant tissue. The residual tissue after thyroid surgery is usually minimal; the detected uptake at a single point (at the 24-h time point) is usually negligible (unless a large remnant is in-situ). This has resulted in the American Thyroid Association (ATA) guidelines suggesting a dose range (derived empirically, ranging from 1100 MBq to as high as 3700 MBq depending on the size of the remnant)^6^. However, the high success rates in ablating remnant thyroid tissue imply that many patients are still treated with higher I-131 doses than required, with potential side effects, such as radiation sialadenitis^6^. In the era of precision medicine, individualized dose estimation is imperative to lower risks and maximize benefits^16^.

There are some limitations to our study. First, it has a small study size (n = 2 for thyrotoxicosis and n = 2 for DTC) as a proof of principle concept. A more extensive prospective clinical study to demonstrate the accuracy and value of clinical outcomes needs to be conducted to examine its clinical applicability. Nevertheless, this is a unique showcase of available technologies to further the cause of individualized medicine. Second, the detectors in the left and right lobe are in proximity, and some scattering of signals from one side of the neck to the other side is highly probable. Since COTI is self-monitored, there might have been variations in the placement around the neck, causing some error in count measurements. Third, the LoHI detectors underwent refractoriness at high counts due to crystal saturation.

Nevertheless, this is a first of its kind of study measuring real-time RAI uptake in thyroid cancer patients. Also, the error and uncertainties in small remnant uptake measurements need to be further investigated. In the future, prospective studies will help us make prediction and dosing algorithms, allowing us to calculate RAI dosing in a personalized way by continuously measuring real-time uptake.

## Conclusions

This is a proof of concept study using a real-time measuring device to quantify RAI uptake in thyroid diseases. This small study lays the groundwork for more extensive trials that provide more extensive data and insights to define the most efficient procedures and analyses to calculate optimal RAI doses for individual patients to limit over-or under-treatment.

## Supplementary Information

Supplementary Information 1.

Supplementary Information 2.

Supplementary Information 3.
