# Supplementary material for: Real-time quantitation of thyroidal radioiodine uptake in thyroid disease with monitoring by a collar detection device
Source: Sci Rep. 2021 Sep 16;11:18479. doi: 10.1038/s41598-021-97408-y (PMC8446004; doi:10.1038/s41598-021-97408-y)
Supplement: Supplementary file 1 — Supplementary Information 1. [file 41598_2021_97408_MOESM1_ESM.doc]

Date: 11/15/2007

Principal Investigator: Prasanna Santhanam, MBBS,MD

Application Number: **IRB00157150**

**JHM IRB - eForm A – Protocol**

- **Use the section headings to write the JHM IRB eForm A, inserting the appropriate material in each. If a section is not applicable, leave heading in and insert N/A.**
- **When submitting JHM IRB eForm A (new or revised), enter the date submitted to the field at the top of JHM IRB eForm A.**

***************************************************************************************************

**Personalized Treatment Planning for Radioiodine Therapy of Thyroid Disease**

**Protocol Version 1.0, dated December 17, 2017**

1. **Abstract**

The Collar Therapy Indicator (CoTI), a device placed in cloth collar around the neck resembling a turtle neck sweater collar with a connecting wire and recording box (Fig. 1), has been shown in a single small previously published experience to provide data regarding radioiodine exposure that correlates with conventional methods of measuring I-123 and I-131 uptakes after diagnostic dose administration and/or therapy for thyroid disorders . We hypothesize that the device’s continuous measurement capability will permit more accurate estimates of radiation exposure to thyroid tissue than conventionally employed methods assessing fractional uptake at one or a few time points. It may also provide information about the extent of variability in the absorbed radiation dose among patients with thyroid cancer and hyperthyroidism. By providing more complete information about individual patient’s exposures, it will facilitate more accurate estimation of the administered I-131 dose requirements for both control of hyperthyroidism and thyroid remnant ablation while reducing the need for repeated visits to the clinic for dosimetry measurements. The aims of our project include the following: (1) Compare quantitative imaging-derived thyroid time activity curve to that obtained using the CoTI and to determine the extent to which there is variability in radiation dose predicted using conventional methods to that predicted from measurement of the full Time-Activity Curve (TAC); (2) Evaluate the uptake and clearance kinetics across the 5 patients in each category as proof of principle for a potential larger trial to investigate use of this device in optimizing the administered doses of radioactive iodine to achieve therapeutic goals while minimizing risks of comorbidities, such a post-radioiodine hypothyroidism in patients with Graves disease. (3) Evaluate patient experience, convenience, and discomfort in using the CoTI device with a survey instrument.

1. **Objectives** (include all primary and secondary objectives)

## Primary Objective;

To perform a pilot (exploratory) study using a novel AG Medical Collar Therapy Indicator (CoTI) to evaluate the variability in radioiodine kinetics across patients treated for hyperthyroid Graves disease and postoperative thyroid remnant ablation for thyroid cancer.

- - For Graves disease, after administration of 0.2 mci I-123 and low dose (200 µCi) I-131, to compare conventional 6, 24, 48 and 72-hour fractional thyroidal radioiodine uptakes with single photon emission (with low-dose computed tomography [SPECT/CT] for quantification and attenuation correction) with the activity measured continuously by the COTI device, which is placed in collar around the neck resembling a turtle neck sweater collar with a wire and recording box.
  - For remnant ablation, after therapeutic administration of 30 mCi I-131, to compare conventional 24, 48, 72 hr. and 7-day SPECT (with low dose CT for quantification and attenuation correction performed at 24, 48, 72 hours and 7 days) with the activity continuously measured by the COTI device, as described above.

**Secondary Objectives;**

## To validate use of the cervical collar device as an enhancement for quantitative measurement of thyroid uptake in patients after diagnostic I-123 and low dose I-131 in Graves disease

## To validate use of the cervical collar device as an enhancement for quantitative measurement of thyroid uptake in patients after therapeutic I-131 for thyroid remnant ablation in thyroid cancer.

## To obtain detailed early-phase thyroid uptake data using a cervical collar device in the Graves disease and thyroid cancer patients as described above.

1. **Background**

Radioiodine treatment for thyroid disorders represented an early example of individualized medicine. Since its introduction 80 years ago, the therapeutic I-131 dosage has usually been tailored to individual patient requirements based on the uptake of a tracer radioiodine dose . Various techniques have been employed to estimate the administered dose of I-131 for optimal therapeutic benefit . Estimated exposure has typically been extrapolated from the results of activity measurements at one or two time points, e.g., at 4 and 24 hours . We now know that treatment of hyperthyroid Graves disease with these methods lead to a 13-25% rate of failure to cure hyperthyroidism and a 46-80% rate of long-term hypothyroidism in cured patients . Similarly, such relatively crude dosimetry doubtless result in significant overtreatment in the case of remnant ablation for differentiated thyroid cancer.For patients with differentiated thyroid cancer requiring remnant ablation, the administered activity has, in most cases, been derived empirically, ranging between 30-100 mci . The high success rates in ablating remnant thyroid tissue implies that many patients are still treated with higher 131-I doses than required, with potential side effects, such as radiation sialadenitis.

1. **Study Procedures**

This will be an unmasked prospective pilot study involving patients with Graves disease (Group I) and differentiated thyroid cancer (Group II). Five patients in each group will have radiation activity measurements using the COTI devise. All persons in the study will receive 131-I treatment based on the current standards of care for dose estimation.

**Study Device;**

The CoTI has 3 components;(Image below)

- A collar unit: a gamma detector wrapped around a body part-neck in this case and contains a scintillating crystal and a silicon photomultiplier. The photon pulse signal is then converted to digital signals (measuring the counts per second). Usually two detectors are placed in specified positions.

- A cable that connects the collar unit to the Control Unit

- A control unit that collects the signals and transmits it wirelessly to a hand-held computer tablet.

Two types of collar devices will be used: the lower activity and the medium activity

1. Lower activity collar for patients with Graves disease as well as diagnostic I-123 scan in thyroid cancer patients. These patients typically receive 0.2-1.2 m Ci of I-123/I-131.
2. Medium activity collar for Patients with thyroid cancer after 131-I ablation, for which patients typically receive 30-100 mci of I-131.

Figure 1.


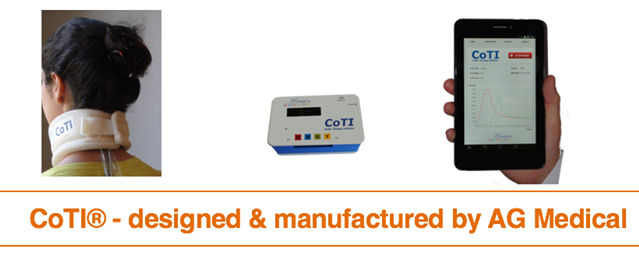


**Study Population;**

- Group I (A,B): Patients with diffuse toxic goiter (Graves disease) diagnosed clinically with no prior history of radioiodine treatment.
- Group II (C ): Patients with differentiated epithelial thyroid cancer who are s/p total thyroidectomy and candidates for remnant ablation based on ATA guidelines.

**IRB approval and HIPPA regulations;**

Since it is a project involving a medical device which will be applied to the patients’ neck, it will require the following;

1. IRB regular approval.
2. Registry into the clinicaltrials.gov.
3. Appropriate forms and consent process for the patients.

**The recruitment of subjects for the study;**

- Informed consent including the benefits and the risks of the Collar device (CoTI) will be obtained.
- There will be separate consent forms for Graves disease and Thyroid Cancer
- Demographic, clinical and laboratory data of the participants will be obtained, as outlined below at the time of placement of the coTI The instructions on the application CoTI will be provided in detail.

Patients would be provided with contact information in the event of any discomfort or challenges while applying the device

Patients would be provided with contact information in the event of any discomfort or challenges while applying the device

The scheduled list of procedures is outlined for the study participants:

1. Group I(A)-Graves disease (I-123 scan)- Table A
2. Group I(B)-Graves disease (I-131 scan)-Table B
3. Group II(C )-Thyroid Cancer (I-131 scan)-Table C

The schedule is at the end of the protocol

**FDA Approval and requirements;**

***The study would qualify for the Investigational Drug Exemption (IDE) regulations based on the published FDA criteria:***

[https://www.fda.gov/MedicalDevices/DeviceRegulationandGuidance/HowtoMarketYourDevice/InvestigationalDeviceExemptionIDE/ucm046164.htm#pre_ide](https://www.fda.gov/MedicalDevices/DeviceRegulationandGuidance/HowtoMarketYourDevice/InvestigationalDeviceExemptionIDE/ucm046164.htm#pre_ide)

CoTI device doesn't deliver energy to the patient nor generate excessive heat. The LoHi detectors will warm up a bit (~30°C/86°F) if  forced on constantly, as in during safety testing, but remain just a few degrees above room temperature in normal use (as per the AG Medical Safety assessment)

**Preparing the patient;**

**Group I (A)-Graves Disease**

The patients will be prepared by general standards of care prior to CoTI placement. For patients with Graves disease (Group IA), any thionamide antithyroid meds will be stopped 3-5 days prior to I-123 diagnostic dose administration (according to the current standard of care). After obtaining written consent, a venous blood sample will be obtained for work for requisite laboratory evaluation for baseline labs(standard of care procedures), as outlined below, and in women, for a serum pregnancy test. A low activity CoTI device will be placed under supervision and then adjusted for comfort and optimal activity measurement as a trial and then removed. Background activity of the patient, a phantom, and the device itself will be measured. After administration of I-123, an initial uptake at time 0 will be obtained. Anatomical localization of the CoTI in all patients will be standardized by identification of the cricoid cartilage, the thyroid cartilage as well as the suprasternal notch.The CoTI device will then be placed and the patient will be asked to return to the clinic at scheduled imaging times for Table A.

**Group I (B) - Graves Disease**

The patients will be prepared by general standards of care prior to CoTI placement. For patients with Graves disease, any thionamide antithyroid meds will be stopped 3-5 days prior to I-131 (0.2 mci) diagnostic dose administration. After obtaining written consent, a venous blood sample will be obtained for work for requisite laboratory evaluation for baseline labs(standard of care procedures), as outlined below, and in women, for a serum pregnancy test. A low activity CoTI device will be placed under supervision and then adjusted for comfort and optimal activity measurement as a trial and then removed. Background activity of the patient, a phantom, and the device itself will be measured. Anatomical localization of the CoTI in all patients will be standardized by identification of the cricoid cartilage, the thyroid cartilage as well as the suprasternal notch in patients with Graves disease, who will be administered 200 µCi of I-131 and fitted with a low-activity CoTI device after administration of the dose. Background activity of the patient, a phantom and the device itself will be measured prior to placement of the device. After administration of I-131, an initial uptake at time 0 will be obtained. The CoTI device will then be placed and the patient will be asked to come back at scheduled imaging times for Table B.

**Group II ( C )- Thyroid Cancer**

Patients with differentiated thyroid cancer will be placed on a low iodine diet for two weeks as per standard of care. Recombinant TSH will be administered on days 1 and 2 (typically Monday and Tuesday), and the diagnostic I-123 dosage will be administered on day 2, as per standard of care. The diagnostic whole-body scan is done on day 3 and the I-131 dose for I-131 ablation (30-100 mci)is administered on day 4, as per standard of care.
After obtaining written consent, venous blood sample will be obtained for work for requisite laboratory evaluation for baseline labs as outlined below, including in women, a pregnancy test prior to the I-131 remnant ablation dose.These are standard of care procedures. The (low to medium activity) CoTI device will be placed under supervision and then adjusted for convenience as outlined above. Background activity of the patient, a phantom, and the device itself will be measured. After administration of I-131, an initial uptake at time 0 will be obtained. Anatomical localization for the CoTI for all patients will be standardized by identification of the cricoid cartilage, the thyroid cartilage as well as the suprasternal notch.The CoTI device will then be placed and the patient will be asked to come back at scheduled imaging times for Group II(Table C).

**Scheduled Imaging times (Tables at the end);**

**Group I (A) - Persons with Graves disease**

After I-123 diagnostic dose;

- An uptake at 0 hours -for background at 9 am

- A planar image with a pinhole collimator at time 06.00 hours with an uptake at the same time.

- An uptake only measurement (with the probe) at time 24.00 hours.

- The counts from the handheld device connected to the CoTI would be obtained at similar times i.e. 0, 6 and 24 hours.The total duration of each visit would be an hour.

**Group I (B) --Persons with Graves disease**

After administration of 200 µCi of I-131 dose;

- A first SPECT image would be performed at 6 hours after I-131 dose.

-A Second SPECT image (with a low dose CT for quantification and attenuation correction) would be performed at 24 hours after I-131 dose.

-A third SPECT image at 48 hours.

-A fourth SPECT image at 72 hours.

- The counts from the handheld device connected to the CoTI would be obtained at similar times as above with one additional time point of 96.00 hours i.e. 6, 24, 48, 72 and 96.

**Group II - Persons with Thyroid Cancer**

- A SPECT image would be performed at 24 hours (with low dose CT for quantification and attenuation correction) after I-131 therapy.

- A second SPECT image at 48 hours.

- A third SPECT image at 72 hours (with low dose CT for quantification and attenuation correction).

A final SPECT CT image (with low dose CT for quantification and attenuation correction) at day 7 post therapy.

- The counts from the handheld device connected to the CoTI would be obtained at similar times as above with one additional time point of 96.00 hours i.e. 24, 48, 96 and day 7.

**Data collection, follow up;**

Baseline data will include age, gender, BMI; duration of autoimmune thyroid disease and/or differentiated thyroid cancer, History/details of thyroid cancer (stage, FNA findings, final tumor histopathology, presence of lymph node metastasis thyroglobulin levels- baseline and stimulated, preoperative ultrasound findings, extent of surgery); presence of co-morbidities, details of autoimmune thyroid disease (prior anti-thyroid regimen including duration of discontinuation, thyroid function tests (free thyroxine, triiodothyronine, TSH), and thyroid stimulating immunoglobulins ), duration and history of levothyroxine therapy (in cases with thyroid cancer), results of neck and thyroid ultrasound, measured neck circumference prior to application of device, quality of life scores as measured by a questionnaire. Imaging data would be acquired as outlined above.

1. Study duration and number of study visits required of research participants.

**The study duration for an individual participant is 10 days and for the overall study would be 2 years but hopefully will be completed in 6 months depending on the ease of recruitement of patients.**

1. Blinding, including justification for blinding or not blinding the trial, if applicable.

**Since it is a device placed around the neck, it cannot be performed in a blinded way. Also, it is a pilot study done with the device to gather preliminary data**

1. Justification of why participants will not receive routine care or will have current therapy stopped.

**Participants will get their care according to current standards for thyroid cancer as well as Graves disease treatment**

1. Justification for inclusion of a placebo or non-treatment group.

**There is no placebo group.**

1. Definition of treatment failure or participant removal criteria.

**Participant will be removed if he is unable to use the device for the stipulated period of time. The data in those cases might be insufficient.**

1. Description of what happens to participants receiving therapy when study ends or if a participant’s participation in the study ends prematurely.

**The participants will be followed up in our practice for visits as determined by their medical condition.**

1. **Inclusion/Exclusion Criteria**

**Inclusion criteria(adults age 21-65):**

**Group I**

Patients with Graves disease confirmed by laboratory testing and who are likely

candidates for I-131 ablation therapy.

Patients being cared for at the Johns Hopkins Hospital East Baltimore and Bayview Campuses

Patients able to understand English and able to follow instructions.

**Group II**

Patients with intermediate and high risk differentiated thyroid cancer requiring radioiodine remnant ablation or moderately high dose I-131 for treatment of residual cervical disease.

Patients receiving care at the Johns Hopkins Hospital- Main campus as well as Bayview Hospital.

Persons able to understand simple English and able to follow instructions.

**Exclusion Criteria;**

- Patients with diseases involving cervical spine (e.g., spondylosis and severe degenerative joint disease).

-Recent iodinated contrast exposure within 8 weeks of the study

-Persons with contraindications for radioactive iodine therapy (e.g.,pregnant women)

1. **Drugs/ Substances/ Devices**
2. The rationale for choosing the drug and dose or for choosing the device to be used.

Our overall objective is to provide the optimal dose to each patient. This study will apply a novel “wearable” radiation detection technology to determine continuous cervical measurements over days following tracer radioiodine administration will provide information that permits the tailoring of subsequent therapeutic radioiodine doses more precisely to improve clinical outcomes, as described above.The actual therapeutic dose decisions in this pilot trial, however, will not be based on the collar device measurements. Patients with Graves disease will receive 180-200 µCi I-131 per gram of estimated gland mass based on the conventional method of dose calculation, based on a 24-hour % uptake and gland volume. Most patients with thyroid cancer would receive the 30 mCi dose for remnant ablation prior to the placement of the COTI device, as per the American Thyroid Association (ATA) guidelines.

1. Justification and safety information if FDA approved drugs will be administered for non-FDA approved indications or if doses or routes of administration or participant populations are changed.

**No non-FDA drugs will be administered. The CT scans are expected to be without iodinated contrast agents.**

Date: ____________________

Principal Investigator: _________________

Application Number: ___________

1. **Study Statistics**
2. Primary outcome variable.
3. Secondary outcome variables.
4. Statistical plan including sample size justification and interim data analysis.

This is a pilot study involving 10 patients and hence there are no interim data analysis or conflicts regarding sample size. Descriptive data will be outlined for all the participants tabulating the variables as determined. The intrapatient variability as well as accuracy and precision of the device will be obtained individually for Group I and Group II patients. The correlation (non-parametric) Spearman correlation coefficient will be obtained to assess the relationship between the uptake and findings as obtained by the collar device and conventional methods of uptake measurement.

1. Early stopping rules.

Early Stopping Rules Include but not limited to:

1. Severe, unexplained discomfort with the device. (2) Lack of device tolerability or interference with optimal functioning of daily activity (3) Discomfort after stopping anti-thyroid treatments causing rebound hyperthyrodism
2. **Risks**
3. Medical risks, listing all procedures, their major and minor risks and expected frequency. b Steps taken to minimize the risks.

The following risks are anticipated:

• Pressure sensation and possible skin irritation due to compression of skin around the neck. The patients can remove the collar for a few hours every day to avoid this.

• Transient redness around the neck if the collar is placed too tightly.

• For persons with prior thyroid surgery, the device might give cause an unpleasant rubbing sensation, especially if the surgical scar is very fresh.

• Rarely, it could cause patients to experience discomfort with swallowing, but the patients will be given all the necessary information to prevent this. If the participants feels uncomfortable, he/she can remove the device anytime

• The participants will also receive 1-4 low-dose CT scans. The cumulative radiation doses will be very low. Radiation imposed minimal risk at very low doses.

- The participants will undergo a low dose 0.2 mci I-131 dosing that is not associated with thyroid “stunning” and is much less than the 12 mCI (about 60 times 0.2) that they will receive for theraupetic treatment of graves disease

1. Plan for reporting unanticipated problems or study deviations.

If there are unanticipated problems, there will be a documentation as per the IRB rules and incident report guidelines. If there are severe unanticipated problems, the study would be discontinued.

1. Legal risks such as the risks that would be associated with breach of confidentiality.

Legal risks include;

- Breach of confidentiality
- Damage to device and lack of compensation for it
- Risks associated with performing a CT scan

1. Financial risks to the participants.

The medical cost of injury due to unanticipated events during the course of the study

Data and Safety Monitoring Plan:

Routine and continuous monitoring for human subject safety events will be undertaken by the Principal Investigator. In addition, the PI, working with the study staff, will oversee the progress of the study and review participant data for untoward events that are anticipated or unexpected.

Adverse events will be reviewed promptly and assessed for trends or to isolate risks not previously identified. Any serious adverse events will be communicated to the IRB per JHMI IRB policies.

1. **Benefits**
2. Description of the probable benefits for the participant and for society.

This study will apply a novel “wearable” radiation detection technology to determine continuous cervical measurements over days following tracer radioiodine administration will provide information that permits the tailoring of subsequent therapeutic radioiodine doses more precisely to improve clinical outcomes, as described above.

The project aims to explore a promising technology for individualizing therapy by assessing the individual variability in the I-123/I-131-time activity curves, minimizing the risk of 131-I administration while achieving highest possible cure rates by tailoring the I-131 dose and lower the need for frequent follow up visits -laboratory and imaging evaluation and health care costs overall.

1. **Payment and Remuneration**
2. Detail compensation for participants including possible total compensation, proposed bonus, and any proposed reductions or penalties for not completing the protocol.

The participants will be compensated with a gift card of 25 USD per visit that is part of the research. The standard of care visits will not be compensated. At the end of the 7 days period,the participants will receive a 50 USD for compliance and completion.

There are no penalties for completing the protocol except that the gift cards will have to be returned back.

1. **Costs**
2. Detail costs of study procedure(s) or drug (s) or substance(s) to participants and identify who will pay for them.

The study is being funded by a small grant received by the Thyroid Tumor Center (under the stewardship of Dr Paul Ladenson) that is a subsidiary of the Division of Endocrinology, Department of Medicine, Johns Hopkins Hospital.

|  |  | ***Run-***  ***Up-days***  ***0*** | ***Day 0***  ***9.00 am*** | ***Day 0***  ***3 pm*** | ***Day 1***  ***9 am*** | ***Follow up***  ***Day***  ***7*** |
| --- | --- | --- | --- | --- | --- | --- |
|  |  | ***-4 ± 1*** |  |  |  |  |
| ***Stop anti-thyroid meds*** | | ***X*** |  |  |  |  |
| ***I-123 dose*** | |  | ***X*** |  |  |  |
| ***COTI measurement*** | |  | ***X^0^*** | ***X^0^*** | ***X^0^*** |  |
| ***Uptake Measurement by probe (SOC)*** | |  | ***X*** | ***X*** | ***X*** |  |
| ***I-123 Planar scan*** | |  |  | ***X*** |  |  |
| ***Informed Consent*** | |  | ***X^0^*** |  |  |  |
| ***Review Eligibility*** | |  | ***X^0^*** |  |  |  |
| ***Medical History*** | |  | ***X*** |  |  |  |
| ***Physical Exam*** | |  | ***X*** |  |  |  |
| ***Vital Signs*** | |  | ***X*** |  |  |  |
| ***Clinical Labs*** | |  | ***X*** |  |  |  |
| ***Pregnancy Test-eligible*** | |  | ***X*** |  |  |  |
| ***Place Collar*** | |  | ***X^0^*** |  |  |  |
| ***Give instructions*** | |  | ***X^0^*** |  |  |  |
| ***Questionnaires*** | |  | ***X^0^*** |  |  |  |
| ***Adverse Events*** | |  | ***X^0^*** |  |  |  |
| ***Concomitant***  ***Medications*** | |  | ***X^0^*** |  |  |  |

***Table A Schedule of Visits after 123 dose for Graves disease Patients – All the visits marked with superscript (o)are part of research-Imaging schedule after I-123 dose***

|  | ***Visit***  ***No.*** | ***Run-***  ***up***  ***0*** | ***Day***  ***0***  ***9 am*** | ***Day***  ***1***  ***9 am*** | ***2***  ***9***  ***am*** | ***3***  ***9***  ***am*** | ***4***  ***9***  ***am*** | ***5*** | ***6*** | ***Follow-***  ***up***  ***7***  ***9am*** |
| --- | --- | --- | --- | --- | --- | --- | --- | --- | --- | --- |
| ***Activity*** | ***Days*** | ***-4 ± 1*** | ***0*** | ***1*** | ***2*** | ***3*** | ***4*** | ***5*** | ***6*** |  |
| ***Stop thionamide***  ***anti-thyroid meds*** | | ***X^*^*** |  |  |  |  |  |  |  |  |
| ***I-131 200 µ administration*** | |  | ***X**** |  |  |  |  |  |  |  |
| ***Uptake Measurement*** | |  | ***X^*^*** | ***X**** |  |  |  |  |  |  |
| ***Informed Consent*** | |  | ***X**** |  |  |  |  |  |  |  |
| ***Review Eligibility*** | |  | ***X**** |  |  |  |  |  |  |  |
| ***Medical History*** | |  | ***X**** |  |  |  |  |  |  |  |
| ***Physical Exam*** | |  | ***X**** |  |  |  |  |  |  |  |
| ***Vital Signs*** | |  | ***X**** |  |  |  |  |  |  |  |
| ***Clinical Labs*** | |  | ***X**** |  |  |  |  |  |  |  |
| ***Pregnancy Test*** | |  | ***X**** |  |  |  |  |  |  |  |
| ***Place Collar*** | |  | ***X**** |  |  |  |  |  |  |  |
| ***Give instructions*** | |  | ***X**** |  |  |  |  |  |  |  |
| ***Questionnaires*** | |  | ***X**** | ***X**** |  |  |  |  |  |  |
| ***SPECT ^$^*** | |  | ***X* 3pm*** | ***X^*^*** | ***X**** | ***X^*^*** |  |  |  |  |
| ***COTI measurement^$^*** | |  | ***X^*^ 3pm*** | ***X^*^*** | ***X^*^*** | ***X^*^*** | ***X**** |  |  |  |
| ***Low dose CT for attenuation correction*** | |  |  | ***X^*^*** |  |  |  |  |  |  |
| ***Adverse Events*** | |  | ***X**** | ***X**** |  |  |  |  |  |  |
| ***Medications*** | |  | ***X**** | ***X**** |  |  |  |  |  |  |

***Table B- Imaging schedule for Graves disease low dose I-131 scan, COTI measurement and SPECT CT at 3 pm that day-The schedule marked with superscript are part of the research.This is a research scan.***

| ***SCHEDULE OF PROCEDURES*** | | | | | | | | | | | |
| --- | --- | --- | --- | --- | --- | --- | --- | --- | --- | --- | --- |
|  | ***Visit***  ***No.*** | ***Run-***  ***up***  ***0*** | ***Day***  ***1***  ***Time***  ***9.00 am*** | ***2***  ***9***  ***am*** | ***3***  ***9 am*** | ***4***  ***9 am*** | ***5***  ***9***  ***am*** | ***6*** | ***7*** | ***8*** | ***Day***  ***11*** |
| ***Activity*** | ***Days*** | ***-4 ± 1*** | ***0*** | ***1*** | ***2*** | ***3*** | ***4*** | ***5*** | ***6*** | ***7*** |  |
| ***Low iodine diet*** | | ***-14 +/-1 day*** |  |  |  |  |  |  |  |  |  |
| ***Informed Consent*** | |  | ***X*** |  |  |  |  |  |  |  |  |
| ***Review Eligibility*** | |  | ***X^1^*** |  |  | ***X^1^*** |  |  |  |  |  |
| ***Medical History*** | |  | ***X^1^*** |  |  |  |  |  |  |  |  |
| ***Physical Exam*** | |  | ***X^1^*** |  |  |  |  |  |  |  |  |
| ***Vital Signs*** | |  | ***X^1^*** |  |  | ***X^1^*** |  |  |  |  |  |
| ***Clinical Labs*** | |  | ***X^1^*** |  |  |  |  |  |  |  |  |
| ***Pregnancy Test*** | |  |  | ***X^1^*** |  |  |  |  |  |  |  |
| ***Thyrogen Injection*** | |  | ***X^1^*** | ***X^1^*** |  |  |  |  |  |  |  |
| ***Diagnostic I-123 dose (1 pm)*** | |  |  | ***X^1^*** |  |  |  |  |  |  |  |
| ***Diagnostic WBS (11 am)*** | |  |  |  | ***X^1^*** |  |  |  |  |  |  |
| ***I-131 30 mci (1PM)*** | |  |  |  |  | ***X^1^*** |  |  |  |  |  |
| ***Give instructions*** | |  | ***X^1^*** |  |  |  |  |  |  |  |  |
| ***Questionnaires*** | |  | ***X^1^*** |  |  |  |  |  |  |  |  |
| ***COTI placement*** | |  |  |  |  | ***X*** |  |  |  |  |  |
| ***COTI measurement*** | |  |  |  |  | ***X*** | ***X*** | ***X*** | ***X*** | ***X*** | ***X*** |
| ***SPECT*** | |  |  |  |  |  | ***X*** | ***X*** | ***X*** |  | ***X^1^*** |
| ***Low dose CT for attenuation*** | |  |  |  |  |  | ***X*** |  | ***X*** |  | ***X^1^*** |
| ***Review of Risks***  ***Benefits*** | |  | ***X*** |  |  |  |  |  |  |  |  |
| ***Adverse Events*** | |  | ***X^1^*** | ***X^1^*** |  | ***X^1^*** |  |  |  |  |  |
| ***Concomitant***  ***Medications*** | |  | ***X^1^*** | ***X^1^*** |  | ***X^1^*** |  |  |  |  |  |

***Table C -Thyroid Cancer Patients-The events not marked with a superscript 1 are part of research. The events in red are part of research protocol. Imaging and visit schedule for thyroid cancer***
