# Supplementary material for: Real-time quantitation of thyroidal radioiodine uptake in thyroid disease with monitoring by a collar detection device
Source: Sci Rep. 2021 Sep 16;11:18479. doi: 10.1038/s41598-021-97408-y (PMC8446004; doi:10.1038/s41598-021-97408-y)
Supplement: Supplementary file 2 — Supplementary Information 2. [file 41598_2021_97408_MOESM2_ESM.pdf]

If you are using Epic for this study, fax a copy of the signed consent form to 410-367-7382.

Patient I.D. plate

RESEARCH PARTICIPANT INFORMED CONSENT AND PRIVACY AUTHORIZATION FORM

Protocol Title: Personalized Treatment Planning for Radioiodine Therapy of Thyroid Disease

Graves Disease Consent

Application No.: IRB00157150

Sponsor: Thyroid Tumor Center, Division of Endocrinology, Johns Hopkins

Principal Investigator:
Prasanna Santhanam MBBS, MD
Assistant Professor in Medicine
Division of Endocrinology, Metabolism and Diabetes
Johns Hopkins University School of Medicine
5501 Hopkins Bayview Circle
Asthma and Allergy Center, suite 3 B 73
Baltimore, MD 21224
Phone: 410-550-6023
Fax: 410-367-2042
E mail: psantha1@jhmi.edu

1. What you should know about this study:

- You are being asked to join a research study. This consent form explains the research study and your part in it. Please read it carefully and take as much time as you need. Ask your study doctor or the study team to explain any words or information that you do not understand.
- You are a volunteer. If you join the study, you can change your mind later. There will be no penalty or loss of benefits if you decide to quit the study.
- During the study, we will tell you if we learn any new information that might affect whether you wish to continue to participate.
- If we think your participation in this study may affect your clinical care, information about your study participation will be included in your medical record, which is used throughout Johns Hopkins. Doctors outside of Johns Hopkins may not have access to this information. You can ask the research team to send this information to any of your doctors.
- When Johns Hopkins is used in this consent form, it includes The Johns Hopkins University, The Johns Hopkins Hospital, Johns Hopkins Bayview Medical Center, Howard County General Hospital, Johns Hopkins Community Physicians, Suburban Hospital, Sibley Memorial Hospital and All Children's Hospital.

2. Why is this research being done?

The purpose of this study is to see if an investigational device called the COTI device will help accurately determine the dose of the radioactive iodine (RAI) that needs to be administered for treatment of patient with thyroid conditions.

This research is being done to accurately determine the dose of RAI needed to treat your Graves disease. Currently, based on a single scan, we try to estimate the dose of RAI needed to treat overactive thyroid disease (such as hyperthyroidism,). However, we might be overtreating (giving too much radioactive substance that may not be required) or undertreating (needing a second treatment dose). The device will measure activity in your neck on a continuous basis giving us information that is likely to be more precise that would help us tailor the dose for each patient without over or under treatment. However, we are not basing your own treatment dose decision based on this new device until we find enough evidence. Hence, there will be no benefit to you to join this research study; it may or may not benefit other patients in the future.

The use of the COTI device (AG Medical Collar Therapy Indicator) in this research study is investigational. The word “investigational” means that the COTI device (AG Medical Collar Therapy Indicator) is not approved for marketing by the Food and Drug Administration (FDA).

People with overactive thyroid disorder called Graves’ disease or with thyroid cancer may join.

How many people will be in this study?

We will enroll 5 persons with Graves’ disease.

3. What will happen if you join this study?

If you agree to be in this study, we will ask you to do the following:

- There will be two imaging studies 1) I123 Study (part research and part treatment) 2) I-131 imaging study (only research)

(1) I-123 Study

In this study, you will be asked to stop your thyroid meds- 4 days prior to the day of the study as a part of the regular standard of care treatment scan. On the first day of the study, you will be asked to read this consent form, understand the study design and objectives in a simple language (as explained above), ask questions and clarify if needed and sign this form in the event, you agree to participate. The collar device will be placed in your neck. We will show you how to switch it on, operate it and remove it if needed. After this, we will give you the I-123 pills as part of the regular standard of care. We will get a scan in about 6 hours’ time as well as measure an uptake by the probe.

You will return the next day again for an uptake measurement and turn over the COTI device to us.

(2) I-131 Study

In this study, you will be asked to stop your thyroid meds- 4 days prior to the day of the study like the I-123 study. On the first day of the study, you will be asked to read this consent form, understand the study design and objectives in a simple language (as explained above), ask questions and clarify if needed and sign this form in the event, you agree to participate. We will give you 200 microcurie of I-131, the risk of which is deemed very low and will be factored in when you receive a larger dose. The collar device will be placed in your neck. We will show you how to switch it on, operate it and remove it if needed. You will come to the imaging center daily for the next 4 days for

imaging studies (just like a CT scan called SPECT) that might last 30-45 minutes daily. On the second day, you will undergo a CT scan too involving a low dose radiation -for an additional 5-10 minutes.

Summarizing your participation;

- Your treatment for your Graves' disease will not be affected by your participation in this study.
- You will be asked to wear the COTI device for a day for the I-123 part of the study
- You will be asked to wear the COTI device on your neck for a period of 7-10 days (for the I-131 part of the study) the picture is included below). However, you will be allowed to remove it periodically every 6 hours for 15-20 minutes
- You will be asked to come for SPECT (with or without CT scan) of the neck and chest to the Nuclear Medicine Department 1 time during the next 7 days (for the I-131 study). After 7 days, you will return the device back to us.
- As a part of the study, you will have to come for visits, CT scan and imaging studies (4-5 times in week) that will last 1-2 hours per day (for I-131 part of the study). The whole study will last a maximum of 8 days. You will continue to receive your treatment for your Graves' disease (as the case may be) according to our usual standard of care.
- In addition to your regular treatment, you will receive a small dose of radioiodine I-131 for a second test (about 200 microcurie of I-131) prior to placement of the neck device in the second part of the study.
- We will stop the anti-thyroid medication just like the previous I-123 scan 4 days before the procedure and this might cause some discomfort.

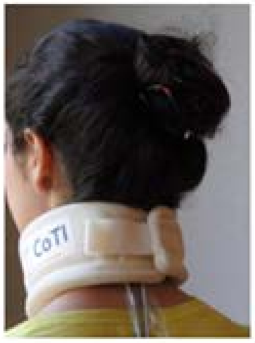

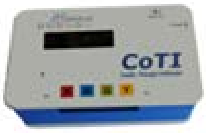

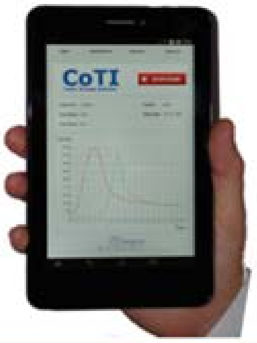

CoTI® - designed & manufactured by AG Medical

The study will not involve any long term follow up.

<u>How long will you be in the study?</u>

You will be in the study for a period of 7-14 days.

4. What are the risks or discomforts of the study?

COTI device:
The risks of placing the device on the neck are as follows:

- Pressure sensation and possible skin irritation due to compression of skin around the neck. You can remove the collar for a few hours every day to avoid that.
- Redness around the neck if you place the collar too tightly.
- For persons with prior thyroid surgery, the device might give cause an unpleasant rubbing sensation especially if the surgical scar is very fresh.
- Rarely, it could cause you to experience discomfort with swallowing, but we will give you all the necessary information to prevent this. If you feel uncomfortable you can remove the device anytime.

Likely: Some local discomfort, inconvenience and discomfort associated with stopping the thyroid medication that includes but not limited to racing heart rate, palpitations, diarrhea.
Less likely: Severe pain, bruising, redness
Unlikely: Choking-like sensation

Standard of care: The risks associated with receiving Radioiodine I-131 0.2 m Ci for Graves' disease and low dose CT scan are reviewed separately as part of treatment.

Radiation:
This research study includes exposure to radiation from x-rays or gamma rays. This radiation exposure is for research purposes only and is not part of your medical care. X-rays and gamma rays can damage cells, but at low doses, the body is usually able to repair these cells.

The radiation exposure that you will get in this research study is 8.118 rem (a rem is a unit of absorbed radiation). To put that in context, the average person in the United States gets a radiation exposure of 0.3 rem per year from natural sources, like the sun, outer space, air, food and soil. People who work with radiation (for example, x-ray technologists) are allowed a maximum exposure of 5.0 rem each year. Although these levels of radiation are thought to cause an increased risk of cancer, studies in people who work with radiation have rarely shown a measurable increase in cancer risk.

The radiation exposure described here is what you will get from this research study only. It does not include any exposure you may have received or will receive from other medical tests outside of this study that are a part of your medical care. If you have questions about the total amount of radiation you will be receiving, you should ask your doctor

You will receive 0.2 mci of I-131 as part of a research scan. This radiation dose will be considered when we are treating your Graves with I-131 when you will receive approximately 70 times this dose.

Questionnaires: You may get tired or bored when we are asking you questions or you are completing questionnaires. You do not have to answer any question you do not want to answer.

Privacy: Wearing the device at home may lead people around you to know that you are part of treatment and/or research.

Unknown Risks: There may be side effects and discomforts that are not yet known.

5. Are there risks related to pregnancy?

If you are pregnant, you cannot receive radiation and so cannot be part of the study.

This research may hurt an embryo or fetus in ways we do not currently know.

6. Are there benefits to being in the study?

There is no direct benefit to you from being in this study. If you take part in this study, you may help others in the future.

7. What are your options if you do not want to be in the study?

If you decide not to join this study, other options are available. You do not have to join this study to get treatment. You will receive the standard of care for Graves' disease.

You do not have to join this study. If you do not join, your care at Johns Hopkins will not be affected.

8. Will it cost you anything to be in this study?

You will receive a separate Insurance and Research Participant Financial Responsibility Information Sheet (Sheet).

This Sheet will give you the following information:

- The procedures, tests, drugs or devices that are part of this research and that will be paid for by the study (no cost to you).
- The procedures, tests, drugs or devices that will be billed to you and/or your health insurer. If you have health insurance, you will be responsible for any co-pays or deductibles not covered by your insurance.

9. Will you be paid if you join this study?

You will receive $25 per visit. You will also receive a parking coupon.
At the end of the study completion, your will receive $50.

You may be required to provide your social security number to be paid for taking part in this study. Federal tax law requires that you report your research payments when you file your taxes. If your total payments from Johns Hopkins exceed $600 per year, Johns Hopkins will report these payments to the Internal Revenue Service and you will receive a 1099-MISC form from us.

10. Can you leave the study early?

- You can agree to be in the study now and change your mind later.
- If you wish to stop, please tell us right away.
- Leaving this study early will not stop you from getting regular medical care.

If you leave the study early, Johns Hopkins may use or give out your health information that it has already collected if the information is needed for this study or any follow-up activities.

11. Why might we take you out of the study early?

You may be taken out of the study if:

- Staying in the study would be harmful.
- You need treatment not allowed in the study.
- You fail to follow instructions.
- You become pregnant.
- The study is cancelled.
- There may be other reasons to take you out of the study that we do not know at this time.

If you are taken out of the study early, Johns Hopkins may use or give out your health information that it has already collected if the information is needed for this study or any follow-up activities**.**

12. How will your privacy be protected?

We have rules to protect information about you. Federal and state laws and the federal medical Privacy Rule also protect your privacy. By signing this form, you provide your permission, called your "authorization," for the use and disclosure of information protected by the Privacy Rule.

The research team working on the study will collect information about you. This includes things learned from the procedures described in this consent form. They may also collect other information including your name, address, date of birth, and information from your medical records (which may include information about HIV status, drug, alcohol or STD treatment, genetic test results, or mental health treatment).

The research team will know your identity and that you are in the research study. Other people at Johns Hopkins, particularly your doctors, may also see or give out your information. We make this information available to your doctors for your safety.

People outside of Johns Hopkins may need to see or receive your information for this study. Examples include government agencies (such as the Food and Drug Administration), safety monitors, other sites in the study and companies that sponsor the study.

We cannot do this study without your authorization to use and give out your information. You do not have to give us this authorization. If you do not, then you may not join this study.

We will use and disclose your information only as described in this form and in our Notice of Privacy Practices; however, people outside Johns Hopkins who receive your information may not be covered by this promise or by the federal Privacy Rule. We try to make sure that everyone who needs to see your information keeps it confidential – but we cannot guarantee that your information will not be re-disclosed.

The use and disclosure of your information has no time limit. You may revoke (cancel) your permission to use and disclose your information at any time by notifying the Principal Investigator of this study by phone or in writing. If you contact the Principal Investigator by phone, you must follow-up with a written request that includes the study number and your contact information. The Principal Investigator's name, address, phone and fax information are on page one of this consent form.

If you do cancel your authorization to use and disclose your information, your part in this study will end and no further information about you will be collected. Your revocation (cancellation) would not affect information already collected in the study, or information we disclosed before you wrote to the Principal Investigator to cancel your authorization.

13. Will the study require any of your other health care providers to share your health information with the researchers of this study?

As a part of this study, the researchers may ask to see your health care records from your other health care providers.

14. What treatment costs will be paid if you are injured in this study?

Johns Hopkins does not have a program to pay you if you are hurt or have other bad results from being in the study. However, medical care at Johns Hopkins is open to you as it is to all sick or injured people.

The costs for any treatment or hospital care you receive as the result of a study-related injury that are not covered by a health insurer will be billed to you.

By signing this form you will not give up any rights you have to seek compensation for injury.

15. What does a conflict of interest mean to you as a participant in this study?

A researcher and Johns Hopkins have a financial or other interest in this study.

In some situations, the results of this study may lead to a financial gain for the researcher and/or Johns Hopkins. This financial interest has been reviewed in keeping with Johns Hopkins' policies. It has been approved with certain conditions, which are intended to guard against bias and to protect participants.

If you have any questions about this financial interest, please talk to Lilja Solnes at 410-955-6989. This person is a member of the study team, but does not have a financial interest related to the study. You may also call the Office of Policy Coordination (410-516-5560) for more information. The Office of Policy Coordination reviews financial interests of investigators and/or Johns Hopkins.

16. What other things should you know about this research study?

a. What is the Institutional Review Board (IRB) and how does it protect you?

The Johns Hopkins Medicine IRB is made up of:

- Doctors
- Nurses
- Ethicists
- Non-scientists
- and people from the local community.

The IRB reviews human research studies. It protects the rights and welfare of the people taking part in those studies. You may contact the IRB if you have questions about your rights as a participant or if you think you have not been treated fairly. The IRB office number is 410-955-3008. You may also call this number for other questions, concerns or complaints about the research.

When the Johns Hopkins School of Medicine Institutional Review Board (IRB) reviews a study at another site, that site (institution) is solely responsible for the safe conduct of the study and for following the protocol approved by the Johns Hopkins IRB.

b. What do you do if you have questions about the study?

Call the principal investigator, Dr. Prasanna Santhanam at cell number 646-281-8111 or office number 410-550-6023. If you wish, you may contact the principal investigator by letter or by fax. The address and fax number are on page one of this consent form. If you cannot reach the principal investigator or wish to talk to someone else, call the IRB office at 410-955-3008.

c. What should you do if you are injured or ill because of being in this study?

If you think you are injured or ill because of this study, call Dr Prasanna Santhanam at 646-281-8111 during office hours.

If you have an urgent medical problem related to your taking part in this study, call Dr Prasanna Santhanam at 410-550-6023 during regular office hours and at 646-281-8111 after hours and on weekends.

d. What happens to Data and Biospecimens that are collected in the study?

Johns Hopkins and our research partners work to understand and cure diseases. The biospecimens and/or data you provide are important to this effort.

If you join this study, you should understand that you will not own your biospecimens or data, and should researchers use them to create a new product or idea, you will not benefit financially.

With appropriate protections for privacy, Johns Hopkins may share your biospecimens and information with our research sponsors and partners.

17. What does your signature on this consent form mean?

Your signature on this form means that: You understand the information given to you in this form, you accept the provisions in the form and you agree to join the study. You will not give up any legal rights by signing this consent form.

WE WILL GIVE YOU A COPY OF THIS SIGNED AND DATED CONSENT FORM

Signature of Participant	(Print Name)	Date/Time

Signature of Physician Obtaining Consent	(Print Name)	Date/Time

I have received the separate Insurance and Research Participant Financial Responsibility Information Sheet.

Signature of Participant, LAR or Parent/Guardian	(Print Name)	Date/Time

NOTE: A COPY OF THE SIGNED, DATED CONSENT FORM MUST BE KEPT BY THE PRINCIPAL INVESTIGATOR; A COPY MUST BE GIVEN TO THE PARTICIPANT; IF YOU ARE USING EPIC FOR THIS STUDY A COPY MUST BE FAXED TO 410-367-7382; IF YOU ARE NOT USING EPIC A COPY MUST BE PLACED IN THE PARTICIPANT'S MEDICAL RECORD (UNLESS NO MEDICAL RECORD EXISTS OR WILL BE CREATED).

ONLY CONSENT FORMS THAT INCLUDE THE JOHNS HOPKINS MEDICINE LOGO CAN BE USED TO OBTAIN THE CONSENT OF RESEARCH PARTICIPANTS.

DOCUMENTATION OF PHYSICIAN/MID-LEVEL PROVIDER CONSENT

My signature below indicates that I have discussed the risks, benefits, and alternatives, answered any questions, and believe the participant is able to make an informed choice to join the study.

____

Signature of Physician/Mid-Level Provider (Print Name) Date/Time

____

Signature of Participant (Print Name) Date/Time

NOTE: A COPY OF THE SIGNED, DATED CONSENT FORM MUST BE KEPT BY THE PRINCIPAL INVESTIGATOR; A COPY MUST BE GIVEN TO THE PARTICIPANT; IF YOU ARE USING EPIC FOR THIS STUDY A COPY MUST BE FAXED TO 410-367-7382; IF YOU ARE NOT USING EPIC A COPY MUST BE PLACED IN THE PARTICIPANT'S MEDICAL RECORD (UNLESS NO MEDICAL RECORD EXISTS OR WILL BE CREATED).

ONLY CONSENT FORMS THAT INCLUDE THE JOHNS HOPKINS MEDICINE LOGO CAN BE USED TO OBTAIN THE CONSENT OF RESEARCH PARTICIPANTS.
